# Assessing the quality of published genetic association studies in meta-analyses: the quality of genetic studies (Q-Genie) tool

**DOI:** 10.1186/s12863-015-0211-2

**Published:** 2015-05-15

**Authors:** Zahra N. Sohani, David Meyre, Russell J. de Souza, Philip G. Joseph, Mandark Gandhi, Brittany B. Dennis, Geoff Norman, Sonia S. Anand

**Affiliations:** Population Genomics Program, Department of Clinical Epidemiology and Biostatistics, McMaster University, Hamilton, ON Canada; Chanchlani Research Centre, McMaster University, Hamilton, ON Canada; Department of Pathology and Molecular Medicine, McMaster University, Hamilton, ON Canada; Department of Medicine, McMaster University, 1280 Main St West, Hamilton, ON L8S 4L8 Canada; Department of Medicine, Western University, London, ON Canada; Programme for Educational Research and Development (PERD), McMaster University, Hamilton, ON Canada

**Keywords:** Quality assessment, Genetic association studies, Genetic epidemiology

## Abstract

**Background:**

Advances in genomics technology have led to a dramatic increase in the number of published genetic association studies. Systematic reviews and meta-analyses are a common method of synthesizing findings and providing reliable estimates of the effect of a genetic variant on a trait of interest. However, summary estimates are subject to bias due to the varying methodological quality of individual studies. We embarked on an effort to develop and evaluate a tool that assesses the quality of published genetic association studies. Performance characteristics (i.e. validity, reliability, and item discrimination) were evaluated using a sample of thirty studies randomly selected from a previously conducted systematic review.

**Results:**

The tool demonstrates excellent psychometric properties and generates a quality score for each study with corresponding ratings of ‘low’, ‘moderate’, or ‘high’ quality. We applied our tool to a published systematic review to exclude studies of low quality, and found a decrease in heterogeneity and an increase in precision of summary estimates.

**Conclusion:**

This tool can be used in systematic reviews to inform the selection of studies for inclusion, to conduct sensitivity analyses, and to perform meta-regressions.

**Electronic supplementary material:**

The online version of this article (doi:10.1186/s12863-015-0211-2) contains supplementary material, which is available to authorized users.

## Background

Completion of the human genome project along with rapid advances in genotyping technology has resulted in an increase in the number of published genetic association studies (Additional file 1: Figure S1) [1].

Systematic reviews and meta-analyses are a common approach to synthesizing these data. However, in combining studies, authors must consider potential limitations and biases introduced by included studies. In addition to the challenges common to classical epidemiological designs (i.e. sampling error, confounding, and selective reporting), genetic association studies face additional unique threats to validity (Table 1). Notably, because a vast majority of genotype-phenotype associations have modest effect sizes, genetic studies must be appropriately powered, often having sample sizes of thousands of subjects. Additional threats to validity include i) quality of genotyping, ii) batch related differences in genotyping, which can manifest as false associations if all cases are in one batch and controls are in the other, iii) choice of inheritance model, and iv) genotype-phenotype relationships confounded by gene-gene and gene-environment interactions [1–3]. Ultimately, inferences from genetic association studies require careful assessment of traditional epidemiologic biases as well as genetic specific threats to validity.

**Table 1:** Common bias in genetic association studies

Bias	Impact on results of genetic association study
Phenotype definition	Unclear definition of phenotype or use of non-standardized definitions can lead to noise in the outcome, which compromises ability to identify corresponding susceptibility variants.
Genotyping misclassification	Differential misclassification of genotypes can positively or negatively affect associations depending on the direction of misclassification. Non-differential misclassification of genotypes will bias association toward the null.
Selection of sample	Source of cases and controls or participants for analysis of quantitative traits can bias the association; for example, contrasting hospital cases with controls from the general population will inflate the association.
Confounding by ethnic origin	If populations from ethnic groups differ in frequency of risk alleles, confounding may occur if the populations are unevenly distributed across comparison groups.
Multiple testing	Testing a multitude of genetic variants against a phenotype creates a possibility of finding significant associations by chance (type 1 error).
Relatedness	Consanguinity in genetic association studies can distort the genotype-phenotype associations. Even in supposed unrelated populations, some individuals may be related. Relatedness should therefore be investigated with additional methods and adjusted for in the statistical analysis.
Treatment effects	The phenotype under investigation may be modified by treatments and hence distort the size of association between genetic variants and the phenotype of interest.

Several guidelines have been published to guide the conduct and reporting of genetic association studies [3–8]. Among the most notable are the Strengthening the Reporting of Genetic Association Studies (STREGA) and Strengthening the Reporting of Genetic Risk Prediction Studies (GRIPS) statements. Furthermore, the Human Genome Epidemiology Network (HuGENet) Working Group developed a grading scheme to aid researchers in assessing the credibility of genetic epidemiological evidence based on three criteria: i) amount of evidence, ii) replication, and iii) protection from bias [2]. Each study is marked as ‘A’, ‘B’, or ‘C’ based on the strength of evidence on the three criteria and a cumulative rating is then obtained using different combinations. While the scheme provides a good baseline to assess evidence in genetic association studies, it is not intuitive to use, and relies on a checklist approach, which has been shown in literature to be less reliable than global rating scales [9]. Moreover, to our knowledge, the grading scheme itself has not been formally tested for validity and reliability.

In this paper, we: i) describe the development of a tool to assess global quality of published genetic association studies, ii) evaluate the tool’s reliability and validity, and iii) investigate whether the reliability and validity of the tool differs based on user’s familiarity with genetic association studies, since there is some evidence to suggest that experts outperform novices on evaluations involving knowledge across different content areas [10–13].

## Methods

### Development of the Q-Genie tool

**Fig. 1:**
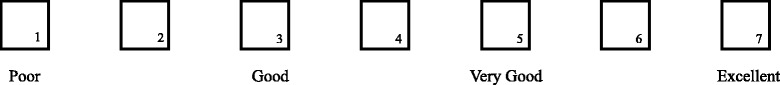
Likert scale used in the Q-Genie tool

Published guidelines and recommendations on appropriate conduct of genetic association studies, including the STREGA and GRIPS guidelines as well as recommendations by *Human Molecular Genetics*, *Diabetologia*, *Nature Genetics,* and individual research groups [3, 5, 7, 8, 14], were used to create a list of items with potential impact on quality. The items were divided into nine categories: rationale for study, selection of sample, classification of exposure, classification of outcome, sources of bias, presentation of statistical plan, quality of statistical methods, testing of assumptions made in genetic studies, and interpretation of results. The categories were then formulated into questions and a description was included to provide context for each question. A Likert type rating scale was created with seven categories anchored by ‘poor’ and ‘excellent’ to ensure minimum loss of precision and reliability and to account for end aversion bias [15]. Additionally, the positive side of the scale was expanded to account for positive skew bias (a tendency to select responses on the favorable end of the scale leading to a ceiling effect in positive ratings) [15]. The final scale used in our tool is depicted in Fig. 1.

A preliminary draft of the tool was sent to five experts with experience in conducting genetic association studies and knowledge in developing measurement tools. The experts were asked to provide suggestions for improvement and comment on the clarity of the items. Discussion with the experts prompted addition of the following aspects lacking from the preliminary draft of the tool: i) checking for samples with outlying heterozygosity, ii) checking both sample and genetic variant missingness, iii) randomization of samples at genotyping stage, iv) checking for concordance of reported sex with genetically determined sex, v) concordance of reported ethnicity with genetically determined ethnicity, and vi) sample size/power considerations. Additionally, the question on classification of the genetic variant was split into two questions, technical and nontechnical classification, respectively.

### Psychometric assessment

We tested the validity and reliability of the Q-Genie tool using a sample of thirty studies randomly selected from a previously conducted systematic review on the association of single nucleotide polymorphisms with type 2 diabetes mellitus in South Asians [16]. Characteristics of the included studies are presented in Additional file 1: Table S1. We used this published systematic review as our sampling frame, instead of a random selection of published studies from scientific databases (e.g. MEDLINE), to ensure generalizability, since the tool is intended for use in systematic reviews.

Four raters, 2 ‘users’ and 2 ‘non-users’, were recruited from the Departments of Clinical Epidemiology & Biostatistics and Medicine at McMaster University. Raters were stratified by user-status, defined as having familiarity with genetic association studies, i.e. if the rater routinely reads/conducts genetic association studies. All four raters each rated the thirty studies for every item of the Q-Genie.

#### Item discrimination

The extent to which each item distinguishes ‘good’ from ‘bad’ quality studies was assessed using item-total correlations. Items with item-total correlations below 0.2 or above 0.9 were considered uninformative and were candidates for exclusion from the tool [15].

#### Reliability

Generalizability theory (G-theory) was used to establish inter-rater reliability (the extent to which a rating from one rater can be generalized to another), internal consistency (the extent to which a rating on one question can be generalized to another), inter-use reliability (the extent to which a rating from users can be generalized to non-users), and overall reliability. Formulas for the coefficients are presented in Additional file 1. All four raters, users and non-users, rated each study. Data from the ratings were used to ascertain G-coefficients, calculated separately for users and non-users, with the exception of inter-user reliability, for which data from both groups were used Raters used in this study were considered a random sample of all possible raters, and therefore we report absolute error G-coefficients.

#### Construct validity

We tested the construct that high quality studies are cited more often and published in higher impact journals. These constructs were evaluated by testing their correlation with total score acquired on Q-Genie. We expected those studies acquiring higher scores on the Q-Genie tool to be published in journals with higher impact factors and cited more often than studies with lower scores on our tool. To account for the fact that some studies were published only in the preceding year and may not have had enough time to be cited, we assessed average citations per year as well as total citations. Additionally, we accounted for self-citation by excluding citations of the paper made by the first and senior authors, as this may artificially inflate the count and bias our assessment of validity. Citation count was ascertained using *Web of Science* (all databases). Correlation was determined using Spearman’s ρ.

### Creating cut-points for low, moderate, and high quality on the Q-Genie tool

In addition to the questions on Q-Genie, raters were given a question on global impression – “rate overall quality of the study”. Ratings of 1 and 2 on this global impression question were classified as ‘low’, 3 and 4 as ‘moderate’, and 5–7 as ‘high’. Borderline groups regression [17], a technique used to establish cut-points, was performed with total score on Q-Genie as the outcome and classification as ‘low’, ‘moderate’, or ‘high’ on the global impression question as the predictor. In this manner, total scores on Q-Genie corresponding to ‘low’, ‘moderate’, and ‘high’ on the global impression question were determined. The global impression question was only used to establish cut-points and is not part of Q-Genie.

### Empirical evaluation of the Q-Genie tool

In addition to the psychometric assessment, we performed an empirical evaluation of the tool using published data from a meta-analysis investigating the association of *CDKAL1* rs7754840 with type 2 diabetes [16]. Meta-analysis of this SNP contained significant heterogeneity and included seven datasets from six studies, making it conducive to this exercise. Characteristics of these studies are presented in Additional file 1: Table S2. We rated all six studies included in the meta-analysis of *CDKAL1* rs7754840 using the Q-Genie tool. If the tool performed as anticipated, the effect estimate for this SNP should be more precise and less heterogeneous after exclusion of low quality studies, determined by Q-Genie, compared to the summary estimate ascertained using all studies. The *I*^*2*^ statistic and Chi square test were used to establish heterogeneity.

Reliability analyses were conducted using G String IV (version 6.1.1). All other analyses were conducted on R (version 3.0.2) and SPSS (version 20.0.0).

## Results

### Description of the final tool

The final version of the Q-Genie tool contained 11 items (i.e. questions) marked on a 7-point Likert scale covering the following themes: scientific basis for development of the research question, ascertainment of comparison groups (i.e. cases and controls), technical and non-technical classification of genetic variant tested, classification of the outcome, discussion of sources of bias, appropriateness of sample size, description of planned statistical analyses, statistical methods used, test of assumptions in the genetic studies (e.g. agreement with the Hardy Weinberg equilibrium), and appropriate interpretation of results. The tool took approximately 20 min to complete per study.

#### Psychometric assessment

##### Item discrimination

Item-total correlations (ITC) were calculated to determine the discrimination of each item (Tables 2 and 3 for users and non-users, respectively). As previously described, an ITC below 0.2 or above 0.9 are generally understood to be uninformative and the corresponding items are considered for exclusion [15]. Overall, no item had an ITC below 0.2 or above 0.9 for either group. The item with the lowest ITC (0.38) for users was question 2, which asked to “rate the study on the classification of the outcome (e.g. disease status or quantitative trait)”. In contrast, question 1 had the lowest ITC for non-users (0.43).

**Table 2:** Item-total correlations and Cronbach’s α if deleted for users

Item	Question	Item-total correlation	Cronbach’s α if item is deleted
Question 1	Please rate the study on the adequacy of the presented hypothesis and rationale.	0.53	0.94
Question 2	Please rate the study on the classification of the outcome (e.g. disease status or quantitative trait).	0.38	0.94
Question 3	Please rate the study on the description of comparison groups (e.g. cases and controls).	0.51	0.94
Question 4	Please rate the study on the technical classification of the exposure (i.e. the genetic variant).	0.86	0.92
Question 5	Please rate the study on the non-technical classification of the exposure (i.e. the genetic variant).	0.55	0.94
Question 6	Please rate the study on the disclosure and discussion of sources of bias.	0.57	0.94
Question 7	Please rate whether the study was adequately powered.	0.84	0.93
Question 8	Please rate the study on description of planned analyses.	0.85	0.92
Question 9	Please rate the study on the statistical methods.	0.87	0.92
Question 10	Please rate the study on the description and test of all assumptions and inferences.	0.80	0.93
Question 11	Please rate the study on whether conclusions drawn by the authors were supported by the results and appropriate methods.	0.88	0.92

**Table 3:** Item-total correlations and Cronbach’s α if deleted for non-users

Item	Question	Item-total correlation	Cronbach’s α if item is deleted
Question 1	Please rate the study on the adequacy of the presented hypothesis and rationale.	0.43	0.90
Question 2	Please rate the study on the classification of the outcome (e.g. disease status or quantitative trait).	0.53	0.89
Question 3	Please rate the study on the description of comparison groups (e.g. cases and controls).	0.51	0.89
Question 4	Please rate the study on the technical classification of the exposure (i.e. the genetic variant).	0.72	0.88
Question 5	Please rate the study on the non-technical classification of the exposure (i.e. the genetic variant).	0.56	0.89
Question 6	Please rate the study on the disclosure and discussion of sources of bias.	0.63	0.89
Question 7	Please rate whether the study was adequately powered.	0.76	0.88
Question 8	Please rate the study on description of planned analyses.	0.55	0.89
Question 9	Please rate the study on the statistical methods.	0.58	0.89
Question 10	Please rate the study on the description and test of all assumptions and inferences.	0.43	0.90
Question 11	Please rate the study on whether conclusions drawn by the authors were supported by the results and appropriate methods.	0.84	0.88

**Fig. 2:**
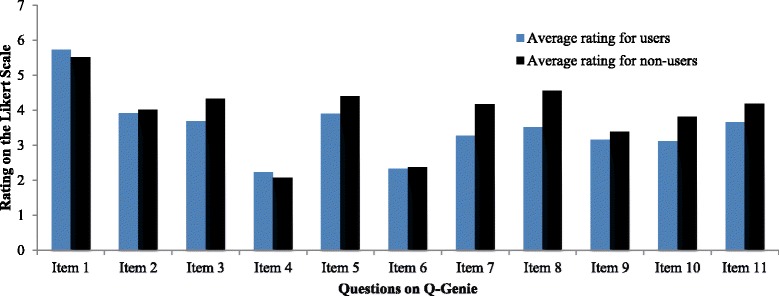
Endorsement of items on the Q-Genie tool in users and non-users

A distribution of average ratings by group for each item is presented in Fig. 2. From the 11 items, item 1, which asked the rater to rate the study on the adequacy of the presented hypothesis and rationale, had the highest endorsement, understood as a rating of 6 or 7 on the 7-point scale, for both groups. On average, users endorsed this item 78 % of the time and non-users endorsed it 60 % of the time. Normally, high endorsement of a question may suggest that the question is not providing discriminative information about each study, since all studies tend to perform well on the item. We did not, however, exclude item 1 from our tool as it provides evidence of face validity and had an acceptable ITC in both groups.

##### Reliability

Analysis of reliability was conducted using G-theory. Inter-rater reliability, internal consistency, and overall reliability were assessed for users and non-users. Inter-rater reliability was 0.74 and 0.45 for users and non-users, respectively. Internal consistency was similar in both groups (G-coefficient of 0.82 in users and 0.80 in non-users). Agreement between users and non-users was 0.64. Lastly, overall reliability, across raters and items, was 0.64 for users and 0.42 for non-users (Table 4).

**Table 4:** G-coefficients of reliabilities, stratified by user-status

Reliability	Users (n_studies_ = 30; n_raters_ = 2)	Non-users (n_studies_ = 30; n_raters_ = 2)
Internal consistency	0.82	0.80
Inter-rater	0.74	0.45
Overall	0.64	0.42
Inter-user*	0.64	

*all four raters were used to estimate this coefficient.

##### Validity

Spearman’s ρ for correlation between impact factor, average citations per year, and total citations, with total score on the Q-Genie tool are presented in Table 5. User scores had a stronger correlation with impact factor and average citations per year than non-user scores, although all values were above ρ = 0.30. Total citations to date had the weakest correlation with scores on Q-Genie for both users and non-users (Spearman’s ρ = 0.40 and 0.33 for users and non-users, respectively), likely because total citations are confounded by time since publication. Spearman’s ρ did not change for either users or non-users when self-citations were excluded from the citation counts.

**Table 5:** Spearman’s ρ correlations of total scores on Q-Genie with impact factor and citation count, stratified by user-status

Construct	Users (n_studies_ = 30; n_raters_ = 2)	Non-users (n_studies_ = 30; n_raters_ = 2)
Impact factor	0.61 (p < 0.01)	0.45 (p = 0.02)
Average citations per year	0.51 (p < 0.01)	0.38 (p = 0.04)
Average citations (without self-cites) per year	0.52 (p < 0.01)	0.39 (p = 0.03)
Total citations to date	0.40 (p = 0.03)	0.33 (p = 0.08)

### Classification as low, moderate, or high quality from total score

**Fig. 3:**
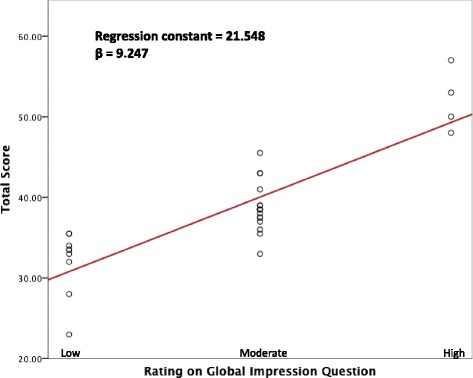
Plot of borderline groups regression depicting total scores on the Q-Genie tool corresponding with ‘low’, ‘moderate’, and ‘high’ quality of genetic association study

Borderline groups regression analysis indicated use of the following cut-points to designate low, moderate, and high quality studies for studies with case/control status as the outcome of interest: scores ≤35 on the Q-Genie tool indicate poor quality studies, >35 and ≤45 indicate studies of moderate quality, and >45 indicate good quality studies (Fig. 3). Similarly, cut-points for studies without control groups (e.g. studies of quantitative traits) were created by excluding question 3 from the calculation of the total score on Q-Genie, since this question asked raters to assess the control group: scores ≤32 on the Q-Genie tool indicate poor quality studies, >32 and ≤40 indicate studies of moderate quality, and >40 indicate good quality studies. Applying these criteria to our sample of 30 studies revealed that 8 out of 30 studies were of poor quality (27 %), 17 were of moderate quality (56 %), and 5 were of high quality (17 %). Of the poor quality studies, a majority had biased technical and non-technical classification of the genetic variant (50 % had a score <3 and 100 % had a score <3 on the respective question), inadequate disclosure of potential sources of bias (100 % had a score <3), inappropriate power (88 % had a score <3), poor statistical methods (75 % had a score <3), and inadequate testing of inferences (63 % had a score <3).

### Empirical evaluation

**Fig. 4:**
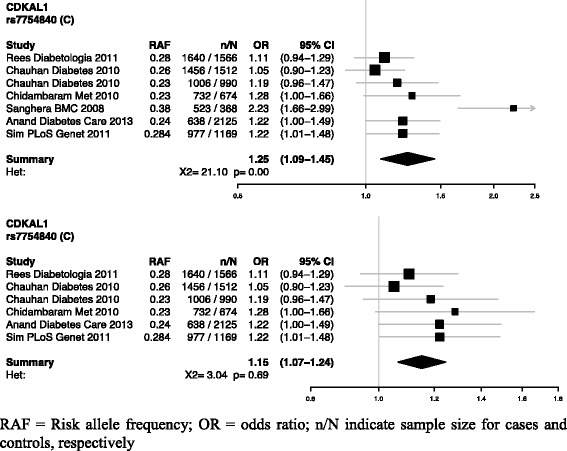
Forest plot of *CDKAL1* rs7754840 with and without exclusion of low quality studies

We applied the Q-Genie to an existing published meta-analysis of *CDKAL1* rs7754840 [16]. After excluding studies with a score of ≤35 on the Q-Genie tool, (those studies deemed to be of poor quality), the heterogeneity in meta-analysis was reduced from an I^2^ of 72 % (95 % CI 38 %–87 %), Q-statistic of 21.1 (6 d.f.; p < 0.01) to an I^2^ of 0 % (95 % CI 0 %–75 %), Q-statistic of 3.04 (5 d.f.; p = 0.69) (Fig. 4). The summary effect size changed from 1.25 (95 % CI 1.09–1.45) to 1.15 (95 % CI 1.07–1.24). Although the difference between the two effect sizes is not statistically significant, the meta-analysis estimate after exclusion of the low quality study had tighter confidence intervals and is more precise.

## Discussion

We have developed and validated a tool that assesses the global quality of published genetic association studies. The Q-Genie can be used to assess quality of genetic association studies in systematic reviews and meta-analyses, which can inform selection of studies for inclusion, examine the sensitivity of pooled effect sizes to indicators of study quality, and/or explain heterogeneity. The tool demonstrated good performance characteristics in a small sample of studies. Additionally, we applied our tool to a published systematic review of studies and found a decrease in heterogeneity and an increase in precision of estimates when used to exclude low quality studies.

### Validity and Reliability

We assessed the validity of the Q-Genie tool by measuring the correlation between predefined constructs, specifically impact factor, citations per year, and total citations with the total Q-Genie score, using Spearman’s ρ. Our findings suggest that Q-Genie demonstrates construct validity in both groups, using measures of impact as a criterion. Other forms of validity should be tested in the future, including predictive, concurrent, as well as convergent and discriminative validity.

We used G-coefficients to estimate the reliability of the Q-Genie tool, which have previously been used to test other instruments in the psychometric literature [18]. Our results show that Q-Genie is highly reliable in users (defined as those who read/conduct genetic association studies frequently) and moderately so in non-users, which is not surprising since users presumably have a better understanding of quality in genetic association studies and are likely to agree more with each other than non-users. Additionally, data from studies in behavioral psychology suggest that people rate individual components based on intuitive impressions from global observations, and thus it appears logical that while different, both user and non-user estimates are reliable [19, 20]. We expect that most users of Q-Genie will be experts in practice.

### Empirical evaluation

When the tool was applied to studies included in a meta-analysis of a well-known SNP associated with type 2 diabetes, we found that by excluding studies graded as poor quality by Q-Genie, we were able to substantially reduce heterogeneity and increase precision of the summary estimate. This furthers our confidence in the utility of the tool for use with systematic reviews and meta-analyses. Use of the tool may also encourage authors to explore other sources of heterogeneity, such as genetic heterogeneity, gene-environment interactions, and gene-gene interactions, if the possibility of between-study heterogeneity due to low-quality data is eliminated.

### Limitations

There are some limitations to our tool. Firstly, data from the four reviewers suggests that the tool takes approximately 20 min per study to complete, slightly longer for non-users (mean of 21 min, 15 s) than users (mean of 18 min, 45 s). Therefore, rating the quality of 30 studies included in a systematic review using Q-Genie would take about 10 h. However, this estimate is comparable with time-to-complete measures of other tools in the literature, such as the Newcastle-Ottawa Scale and AMSTAR [21–23]. Additionally, once accustomed to the tool, raters likely become faster. Though the procedure may be time intensive, the gains in scientific rigor appear well worth the effort as demonstrated by application to the systematic review of the *CDKAL1* SNP. Secondly, as with other global scoring tools, it is possible for a study to receive low scores on 2 dimensions, yet high scores on all others, and thus be considered a globally ‘high quality’ study. This can have limitations for answering specific research questions. However, because it is possible to obtain a score on each section using Q-Genie, users can be mindful of performance on each dimension. Lastly, because this is a pilot assessment with 30 studies and 4 reviewers, additional testing is warranted to gain support for our findings.

Q-Genie is available for download from <http://fhs.mcmaster.ca/pgp/links.html>. We note that our tool would benefit from testing in a larger sample of studies as well as an assessment of additional measures of validity, and we encourage other groups to further test our tool. We also welcome comments that can be used to inform revisions of the tool.

## Conclusions

Based on our evaluation of 30 studies from a published systematic review, it appears that many publications in literature may be of poor quality despite published guidelines designed to improve the quality of genetic association studies. The Q-Genie tool was developed and validated to facilitate the assessment of published studies and to ultimately identify high quality studies when planning meta-analyses of genetic association studies. Integration of our tool into systematic reviews and meta-analyses can help improve the state of evidence in the field of genetic epidemiology, which is currently plagued with irreproducible findings. Our data shows that Q-Genie demonstrates good inter-rater reliability, internal consistency, and overall reliability. We encourage using the Q-Genie tool as it can substantially increase the quality of meta-analyses in genetic association studies.

## Additional file

Additional file 1:
**Supplementary Box 1 Formulas for absolute error G-coefficients for reliability.** **Figure S1.** Frequency plot of increase in publication of genetic association studies (determined via a search of PubMed). **Table S1.** Description of studies used for psychometric assessment of Q-Genie. **Table S2.** Description of studies included in the meta-analysis of *CDKAL1* rs7754840 [16] used in the empirical evaluation of Q-Genie.
