# Supplementary material for: Assessing the quality of published genetic association studies in meta-analyses: the quality of genetic studies (Q-Genie) tool
Source: BMC Genet. 2015 May 15;16:50. doi: 10.1186/s12863-015-0211-2 (PMC4431044; doi:10.1186/s12863-015-0211-2)
Supplement: Additional file 1: — Supplementary Box 1 Formulas for absolute error G-coefficients for reliability. Figure S1. Frequency plot of increase in publication of genetic association studies (determined via a search of PubMed). Table S1. Description of studies used for psychometric assessment of Q-Genie. Table S2. Description of studies included in the meta-analysis of CDKAL1 rs7754840 [16] used in the empirical evaluation of Q-Genie. [file 12863_2015_211_MOESM1_ESM.docx]

**Supplementary Box 1** Formulas for absolute error G-coefficients for reliability

| G-coefficients were calculated as follows:  $G= \frac{\sigma^{2}\left( \tau\right)}{\sigma^{2}\left( \tau\right)+\sigma^{2}\left( \Delta\right)}$ ;  where **τ** contains the variance due to the facet of differentiation (the studies) and all variances and interactions associated with fixed facets and **Δ** contains any variance component with any random facet in it (including interactions between random and fixed facets) and any main effects associated with the random effects  The random and fixed facets are defined as follows for the various G-coefficients:   \| **G-coefficient** \| **Fixed** \| **Random** \| \| --- \| --- \| --- \| \| Inter-rater reliability \| Items \| Raters \| \| Inter-question (internal consistency) \| Raters \| Items \| \| Overall reliability \|  \| Items, Raters \| \| Inter-user \| Items, Raters:User status \| User status \| |
| --- | --- | --- | --- | --- | --- | --- | --- | --- | --- | --- | --- | --- | --- | --- | --- |

**Supplementary Figure 1** Frequency plot of increase in publication of genetic association studies (determined via a search of PubMed)

**Supplementary Table 1** Description of studies used for psychometric assessment of Q-Genie

| **Authors** | **Year of publication** | **Population studied** | **Source of cases** | **Source of controls** | **Sample size (cases/controls)** |
| --- | --- | --- | --- | --- | --- |
| Bodhini *et al.* (a) | 2011 | South Indians | CURES cohort: general population | CURES cohort: general population | 643/552 |
| Tan J *et al.* | 2010 | Singaporean Indians | SDCS: recruited from Singapore National Healthcare Group Polyclinics; NHS98: general population | NHS98: general population | 246/364 |
| Bodhini *et al.* | 2011 | South Indians | CURES cohort: general population | CURES cohort: general population | 649/794 |
| Haseeb *et al.* | 2009 | South Indians | Recruited from Mediciti hospital | Recruited from Mediciti hospital | 350/349 |
| Ganasyam *et al.* | 2012 | South Indians | Recruited from Institute of Genetics and Hospital | Age and sex matched, recruited from Institute of Genetics and Hospital | 100/100 |
| Gupta *et al.* | 2011 | North Indians | Endocrinology outpatient of the Nehru hospital | Healthy individuals attending a general medical clinic for health check up | 250/300 |
| Vimaleswaran *et al.* | 2010 | South Indians | CURES cohort: general population | CURES cohort: general population | 810/990 |
| Nair *et al.* | 2010 | South Indians | ADAWS cohort: endocrinology department at Amrita Institute of Medical Sciences | ADAWS cohort: medical camps for screening of risk factors and increasing diabetes awareness | 994/984 |
| Dwivedi *et al.* | 2013 | North Indians | Urban regions in and around Delhi | Urban regions in and around Delhi as a part of an ongoing type 2 diabetes case–control study | 2049/2028 |
| Gill *et al.* | 1991 | North Indians | Recruited from local general hospitals | Recruited from local general hospitals | 95/70 |
| Sugunan *et al.* | 2010 | South Indians | ADAWS cohort: endocrinology department at Amrita Institute of Medical Sciences | ADAWS cohort: medical camps for screening of risk factors and increasing diabetes awareness | 1000/1000 |
| Sim *et al.* | 2011 | Singaporean Indians | SINDI study: randomly sampled from general population | SINDI study: randomly sampled from general population | 977/1169 |
| Sanghera *et al.* | 2010 | North Indians | SDS cohort: endogamous Khatri Sikh population | SDS cohort: endogamous Khatri Sikh population with no family history of type 2 diabetes; 262 were non-diabetic spouses | 554/527 |
| Achyut *et al.* | 2007 | North Indians | Recruited from clinics of  Sanjay Gandhi Postgraduate Institute of Medical Sciences | Healthy staff from same hospital | 200/223 |
| Humphries *et al.* | 2006 | South Asians (region not specified) | WHSS: recruited from general practice clinics, PREDICT: recruited from the diabetes clinics, UCS: recruited from the diabetes clinics | WHSS: recruited from general practice clinics | 841/302 |
| Saxena *et al.* | 2012 | North Indians | Outpatient Diabetes clinic | Healthy staff from university hospital | 221/205 |
| Anand *et al.* | 2013 | South Asians (region not specified) | EpiDREAM cohort: Individuals at risk for dysglycemia recruited from 191 centres around the world via a variety of methods | EpiDREAM cohort: sample population as cases | 638/2125 |
| Raza *et al.* | 2012 | North Indians | Recruited from a diabetic clinic | Recruited from same diabetic clinic with no history of type 2 diabetes | 87/88 |
| Bhat *et al.* (a) | 2007 | Kashmiri South Asians | Not specified | Not specified | 199/213 |
| Bodhini *et al.* | 2012 | South Indians | CURES cohort: general population | CURES cohort: general population | 1010/1016 |
| Tai *et al* | 2004 | Singaporean Indians | NHS98 cohort: general population | NHS98 cohort: general population | 108/305 |
| Bhaskar *et al* | 2011 | South Indians | Recruited from hospitals | Not specified | 310/120 |
| Kooner *et al* | 2011 | South Asians (region not specified) | LOLIPOP: recruited from lists of GPs in West London; PROMIS: out-patient departments; SINDI: general population; COBRA: general population; DGP: hospitals or Diabetes awareness camps; CURES: general population; Mauritius cohort: population based survey; RHS: population based survey used to participate in study; SDS: endogamous Khatri Sikh population; SCCS: recruited from hospitals and polyclinics | LOLIPOP: recruited from lists of GPs in West London; PROMIS: matched cases from visitors in out-patient; SINDI: general population; COBRA: general population; DGP: recruited from community screening camps; CURES: random sampling from general population; Mauritius cohort: population based survey; RHS: population based survey; SDS: endogamous Khatri Sikh population with no family history of type 2 diabetes, 262 were non-diabetic spouses; SCCS: general population | 18731/39856 |
| Vimaleswaran *et al* | 2005 | South Indians | CURES cohort: general population | CURES cohort: general population | 515/882 |
| Tabassum *et al* | 2013 | South Asian (both North and South Indians) | INDICO: consecutively recruited from the Endocrinology clinic of All India Institute of Medical Sciences; CURES: general population | INDICO: recruited from diabetes awareness camps; CURES: general population | 6738/ 5797 |
| Bhatti *et al* | 2010 | North Indians | NIDS: general population | NIDS: general population | 328/326 |
| Vimaleswaran *et al* | 2011 | South Indians | CURES cohort: general population | CURES cohort: general population | 487/919 |
| Adak *et al* | 2010 | Bengali Indians | Not specified | Not specified | 200/100 |
| Bhat *et al* (b) | 2007 | Kashmiri South Asians | Not specified | Not specified | 152/258 |
| Rees *et al* | 2008 | Pakistanis | Not specified | Same geographical areas through community screening | 831/437 |

**Supplementary Table 2** Description of studies included in the meta-analysis of *CDKAL1* rs7754840 [16] used in the empirical evaluation of Q-Genie

| **Author** | **Year of publication** | **Population studied** | **Source of cases** | **Source of controls** | **Sample size** |
| --- | --- | --- | --- | --- | --- |
| Rees *et al.* | 2011 | North Indians and Pakistanis | UKADS: not specified; DGP: hospitals in Mirpur | UKADS: same geographic region; DGP: community screening | 1678/1584 |
| Chauhan *et al.* | 2010 | North Indians | Delhi: consecutively recruited from the Endocrinology clinic of All India Institute of Medical Sciences; Pune: general population in Pune and surrounding areas | Delhi: urban dwellers of Indo-European ethnicity with no family history of diabetes in ﬁrst and/or second degree relatives; Pune: parents of children in the Pune Maternal Nutrition Study and Coronary Risk of Insulin Sensitivity in Indian Subjects study | 2486/ 2678 |
| Sanghera *et al.* | 2008 | North Indians | SDS cohort: endogamous Khatri Sikh population | SDS cohort: endogamous Khatri Sikh population with no family history of type 2 diabetes; 262 were non-diabetic spouses | 532/386 |
| Chidambaram *et al.* | 2010 | South Indians | CURES cohort: general population | CURES cohort: general population | 926/812 |
| Anand *et al.* | 2013 | South Asians (region not specified) | EpiDREAM cohort: Individuals at risk for dysglycemia recruited from 191 centres around the world via a variety of methods | EpiDREAM cohort: sample population as cases | 638/2125 |
| Sim *et al.* | 2011 | Singaporean Indians | SINDI study: randomly sampled from general population | SINDI study: randomly sampled from general population | 977/1169 |
